# Use of cell-free signals as biomarkers for early and easy prediction of preeclampsia

**DOI:** 10.3389/fmed.2023.1191163

**Published:** 2023-05-24

**Authors:** Jean Gekas, Theresa Hopkins Boomer, Marc-André Rodrigue, Kristine N. Jinnett, Sucheta Bhatt

**Affiliations:** ^1^Department of Medical Genetics, Quebec University Mother and Child Center, Laval Medical University, Quebec City, QC, Canada; ^2^Illumina, Inc., San Diego, CA, United States

**Keywords:** preeclampsia, cell-free DNA, prenatal screening, fetal fraction, concentration, fragment size distribution, biomarker

## Abstract

**Introduction:**

Preeclampsia (PE) is a leading cause of maternal and perinatal morbidity worldwide. However, current methods of screening are complicated and require special skill sets. In this observational study of prospectively collected samples, we wanted to evaluate if cell-free (*cf*) DNA could be an efficient biomarker for identification of at-risk patients.

**Methods:**

One hundred patients attending a private prenatal clinic in Canada were enrolled in their first trimester of pregnancy and a blood draw was carried out at 11 + 0 to 14 + 2 weeks’ (timepoint A) and 17 + 6 to 25 + 5 weeks of gestation (timepoint B). CfDNA signals, namely concentration, fetal fraction, and fragment size distribution, were correlated with clinical outcomes in the test population to develop the logistic regression model.

**Results:**

Twelve patients developed PE—four early-stage and eight late-stage PE. Significant differences were observed between PE patients and control cases for all three cfDNA signals at timepoint A, while both fetal fraction and concentration were significantly different between PE patients and control cases at timepoint B. Overall, the model had a sensitivity of up to 100% and specificity of up to 87.5% at Timepoint A.

**Conclusion:**

This proof-of-principle study showed that use of this logistic regression model could identify patients at risk of preeclampsia in the first trimester of pregnancy.

## Introduction

Worldwide, preeclampsia (PE) typically affects 2–8% of pregnant women and is one of the leading causes of maternal and perinatal morbidity (1–3). Severe PE can lead to preterm birth, fetal growth restriction, maternal multiorgan dysfunction, maternal seizures, and perinatal death, with around 46,000 women and 500,000 babies dying from this disorder every year (1, 4–6). Preeclampsia can lead to hepatic and hematopoietic dysfunction and can have a significant impact on the renal and nervous systems (6). In addition, mothers that had PE and children from affected pregnancies have an increased risk of long-term cardiovascular disease and chronic diseases, with the life expectancy of women who developed preterm PE being reduced on average by 10 years (4, 7). Preeclampsia is a multifaceted syndrome with a highly variable clinical presentation and can be classified into two main subtypes, namely early-onset PE and late-onset PE. The International Society for the Study of Hypertension in Pregnancy (ISSHP) classify (*de novo*) PE as gestational hypertension accompanied by one of three new-onset conditions at ≥20 weeks of gestation, namely proteinuria, other maternal end-organ dysfunction, or uteroplacental dysfunction (8).

There are a number of risk factors associated with development of PE including advanced maternal age, chronic hypertension, autoimmune diseases such as antiphospholipid antibody syndrome and systemic lupus erythematosus, pregestational diabetes, and multifetal pregnancies (4, 9). Currently, there is no specific treatment for PE, with induction of labor being the only treatment for severe cases. Meta-analyses of randomized trials found that an appropriate daily dose of aspirin initiated before 16 weeks of gestation could reduce the risk of developing PE as well as its related complications (5, 10, 11). The American College of Obstetricians and Gynecologists (ACOG) recommends that women with any of the high-risk factors for PE and those with more than one moderate risk factor should receive low-dose aspirin starting between 12 and 28 weeks of gestation (preferably before 16 weeks’ gestation) until delivery (2). It is therefore optimal that screening for PE is carried out in the first trimester of pregnancy.

There have been a variety of different methods proposed for screening patients for PE during pregnancy. One of the main screening methods currently used is based on the Bayes based competing risk (CR) model. This PE screening method combines maternal characteristics and obstetric history with biomarkers for risk assessment including mean arterial pressure, uterine artery pulsatility index, and placental growth factor (PlGF) (12, 13). Although this method is superior to the traditional approach that is based solely on maternal medical history and demographic characteristics, it is complicated and requires multiple different tests and information for calculating PE risk assessment.

In this proof-of-principle study, we wanted to determine if cell-free (*cf*) DNA could be used as an efficient biomarker for identification of patients at risk of developing PE. The identification of fetal cfDNA in maternal circulation in 1997 (14) has led to a revolution in prenatal screening worldwide. Currently, cfDNA obtained from a maternal blood draw during pregnancy is used extensively for prenatal aneuploidy screening. As cfDNA originates from the developing placenta, it is possible that the release of cfDNA is related to placental size and the rate of placental apoptosis (15–17). As PE is a placental disorder, cfDNA could therefore be a useful biomarker to screen for this condition. Previous studies have noted a relationship between cfDNA-based signals, such as concentration and fetal fraction, and PE in pregnant patients (16, 18–25). Here, we looked at whether cfDNA-based signals at two different timepoints (11 + 0 to 14 + 2 weeks’ gestation and 17 + 6 to 25 + 5 weeks’ gestation) differed between patients that did and did not develop preeclampsia during their pregnancy. We also compared the screening performance of our proposed model to that of the CR model.

## Methods

We conducted an observational study of prospectively collected samples from pregnant patients to determine if cfDNA-based signals could be used to identify patients at risk of developing PE. The test population for our proposed model consisted of samples from pregnant patients attending a private prenatal clinic in Quebec City (Cliniques Prenato, Canada) for first-trimester routine noninvasive prenatal testing (NIPT) for aneuploidy and other adverse obstetrical outcomes. Visits were held at 11 + 0 to 25 + 5 weeks of gestation from July 2020 to May 2021, where nurses took a record of maternal characteristics and medical history including maternal age, gestational age, maternal height and weight, racial origin, if the patient was a cigarette smoker during pregnancy, if there was any PE history in the mother of the patient, and the conception method for the current pregnancy. This was then reviewed by a specialized doctor. Blood was taken for PlGF serum levels, mean arterial blood pressure was measured, as well as left and right uterine artery pulsatility index using a transabdominal color Doppler ultrasonography. Patients were enrolled in the study if they were at least 18 years of age, had a singleton pregnancy, and a gestational age (GA) of 11 + 0 to 14 + 2 weeks. Patients were excluded from the study if they had chronic hypertension, were taking aspirin or any anticoagulant drug, were on immunosuppressors, or either currently or previously had cancer. A blood sample was collected as part of routine NIPT screening process at 11 + 0 to 14 + 2 weeks of gestation (timepoint A) with a repeat blood draw at 17 + 6 to 25 + 5 weeks of gestation (timepoint B) for the purposes of this study. Blood samples from timepoint A were processed using Verifi™ lab developed test, an adapted version of Illumina’s VeriSeq™ NIPT Solution v2 assay (Illumina, Inc. clinical services laboratory, Foster City, CA). Blood samples from timepoint B were run on the RUO version. The NIPT results on rare autosomal trisomies or copy number variations from either timepoint A or timepoint B were not reported to the patient. Only the validated results for trisomy 21, trisomy 18, and trisomy 13 from timepoint A were returned to the patient. All women gave their written consent to participate and share the obtained data in the study. This project has received approval from Prenato Clinics Institutional Research Review Board (Approval Number 12302019–2).

Follow-up was carried out on all study participants until delivery. The neonatal examination report was collected if an adverse outcome was reported. Outcome information included birth weight, GA at delivery, method of delivery, and if there were any delivery complications. Outcomes included PE or delivery with PE and other adverse pregnancy/birth outcomes; PE was defined according to the International Society for the Study of Hypertension in Pregnancy (26).

CfDNA parameters from the NIPT bioinformatics analysis for timepoint A were used in the development of our model. The formula used for the cfDNA model was as follows: y_i_ = β0-β1 (FragSizeDist) + β_2_ (FragSizeDist:FF)– β_3_ (FragSizeDist:FF:Conc) where y_i_ is the dependent or predicted variable of PE; β_0_ = 2.88 (the y-intercept); and β_1_ = 87.19, β_2_ = 745.75, and β_3_ = 8.12 which are the regression coefficients for FragSizeDist, the interaction between FragSizeDist with FF, and the interaction of FragSizeDist with FF and Conc, respectively. FragSizeDist is a summary statistic that captures the standard deviation of the differences between the observed and expected cfDNA fragment size distribution. FF is calculated using information from both the cfDNA fragment size distribution and the differences in genomic coverage between maternal and fetal cfDNA (27). Conc is the library concentration as measured by a fluorescent dye with concentration determined relative to a DNA standard curve.

In the multiple logistic regression model, PE outcome was a dependent binary variable with cfDNA fetal fraction (FF) and fragment size distribution (FragSizeDist), ± cfDNA concentration (Conc), as independent variables. Further, the model predicted the probability of PE based on FragSizeDist as an independent variable, along with interaction terms that include FF and Conc. Supervised fivefold cross validation was performed on the model with a minimum number of PE cases in the training and test sets. Training of the selected model utilized ~80% of patient data (*n* = 75, 8 PE cases +67 non-PE cases) with ~20% of patient data (*n* = 20, 4 PE cases +16 non-PE cases) used in the test set. The supervised 5-fold cross-validation of the model resulted in 90% accuracy.

All bioinformatic statistical analyses were performed using RStudio software program v 2022.07.2 build 576 © 2009–2022.

## Results

A total of 100 samples from pregnant patients were prospectively collected. Five samples did not have outcome data and so were excluded. Twelve patients developed PE, of which four were early-stage (before 34 weeks of gestation) and eight were late-stage PE (after 34 weeks of gestation); no aneuploidies were detected in the group of patients that developed PE. Patient demographics for our test population are shown in Table 1. We have divided the patients into four separate categories, depending on their observed PE outcome and their screen-positive status by the Competing Risk model or our cfDNA signals model. Patients that had any adverse pregnancy outcomes were excluded from the control group. Biomarker levels were measured for all study participants and included blood pressure, uterine artery pulsatility index, and PlGF; details of these for each of the four groups are also shown in Table 1.

**Table 1:** Maternal demographics and pregnancy history of study participants.

Variable	Patients with no PE or APO (*n* = 48)	Patients with observed PE (*n* = 12)	High-risk by CR model (*n* = 17)	High-risk by cfDNA model^a^ (*n* = 6)
Maternal age, yr	30.08 (28.26–31.34)	29.89 (27.65–30.91)	29.31 (27.41–33.93)	30.01 (29.08–30.27)
Maternal height, cm	166.50 (166.00–170.00)	163.00 (155.00–165.00)	163.00 (155.00–165.00)	164.00 (157.00–165.00)
Maternal weight, kg	67.30 (60.23–76.78)	79.45 (62.15–92.91)	73.40 (61.70–82.40)	78.80 (74.98–104.98)
Body mass index, kg/m^2^	24.51 (22.32–27.33)	27.59 (25.84–37.39)	27.56 (23.56–33.24)	32.91 (28.45–39.88)
Gestational age, wks + days	12 + 2 (12 + 1–12 + 4)	12 + 3 (12 + 1–12 + 6)	12 + 2 (12 + 0–12 + 6)	12 + 5 (12 + 3–12 + 6)
Racial origin				
White	47 (98)	12 (100)	16 (94)	6 (100)
Other	1 (2)	0 (0)	1 (6)	0 (0)
Cigarette smokers	1 (2)	1 (8)	0 (0)	0 (0)
Family history of PE	2 (4)	0 (0)	2 (12)	0 (0)
Method of conception				
Natural	47 (98)	11 (92)	15 (88)	6 (100)
Ovulation induction	0 (0)	1 (8)	2 (12)	0 (0)
*In vitro* fertilization	1 (2)	0 (0)	0 (0)	0 (0)
Parity				
Nulliparous	22 (46)	11 (92)	15 (88)	4 (67)
Parous, no previous PE	26 (54)	1 (8)	2 (12)	2 (33)
Parous, previous PE	0 (0)	0 (0)	0 (0)	0 (0)
Interpregnancy interval, yr	1.80 (1.30–2.10)	0.30 (0.30–0.30)^b^	4.65 (3.33–5.98)	2.15 (2.13–2.18)
Biomarker levels as measured				
Systolic blood pressure (mmHg)	105.88 (101.69–113.31)	120.00 (113.38–127.25)	118.25 (115.50–123.75)	121.38 (106.81–126.19)
Diastolic blood pressure (mmHg)	67.75 (65.00–72.63)	80.63 (74.69–81.81)	81.50 (76.50–83.00)	79.50 (69.13–81.44)
Mean blood pressure (mmHg)	81.13 (77.73–85.17)	93.46 (88.85–97.17)	93.92 (89.25–98.25)	93.46 (81.77–96.27)
UAPI	1.42 (1.15–1.54)	1.84 (1.42–2.03)	1.49 (1.37–1.88)	1.80 (1.53–1.88)
PlGF (ng/mL)	21.85 (18.60–28.15)	18.95 (11.68–28.68)	12.10 (6.00–17.10)	29.75 (23.43–30.08)
Standardized biomarker levels				
Mean blood pressure, MoM	0.94 (0.91–1.00)	1.04 (1.02–1.05)	1.05 (1.02–1.09)	1.03 (0.93–1.04)
UAPI MoM	0.87 (0.69–0.97)	1.14 (0.90–1.24)	0.94 (0.86–1.13)	1.18 (0.98–1.21)
PIGF MoM	0.73 (0.58–0.90)	0.63 (0.45–0.93)	0.38 (0.26–0.55)	0.94 (0.72–1.24)

Data are expressed are median (interquartile range) or n (%) unless noted otherwise. APO, adverse pregnancy outcomes; MoM, Multiple of the median; PE, preeclampsia; PlGF, Placental growth factor; UAPI, Uterine artery pulsatility index; wk; week; yr, year.

^a^Based on the test set.

^b^Based on one patient only.

**Figure 1:**
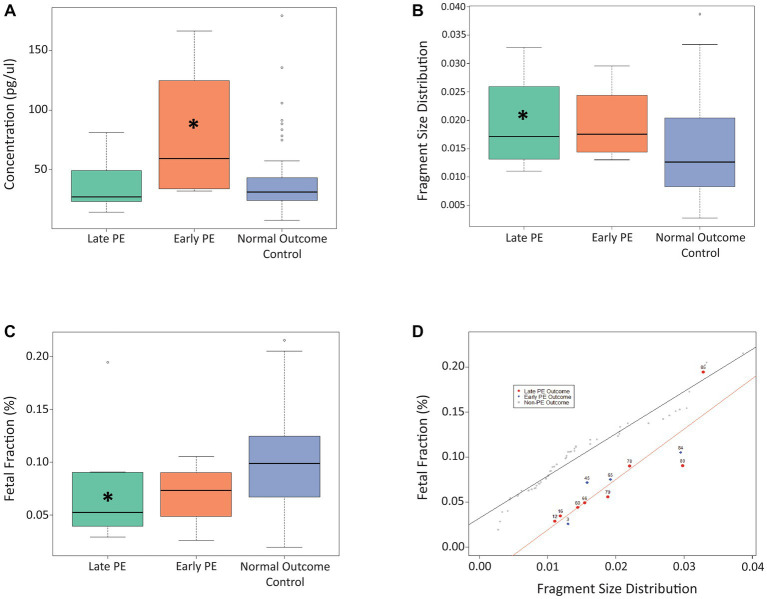
Use of cfDNA signals to distinguish between late PE, early PE, and control samples at timepoint A. **(A)** Use of concentration at timepoint A to distinguish between the three patient cohorts. **(B)** Use of fragment size distribution at timepoint A to distinguish between the three patient cohorts. **(C)** Use of fetal fraction at timepoint A to distinguish between the three patient cohorts. **(D)** Linear regression plot showing the use of fetal fraction and fragment size distribution to distinguish between PE samples and control samples at timepoint A. The Asterix (*) denotes significance between that particular PE group and the control group.

For our proposed model, three different cfDNA signals were considered, namely cfDNA concentration, fetal fraction, and fragment size distribution. Figure 1 shows the impact of these different cfDNA signals on distinguishing cases of early-stage and late-stage PE patients from control samples at timepoint A (11 + 0 to 14 + 2 weeks of gestation). As can be seen from Figure 1A, the concentration of cfDNA from cases with early PE was significantly higher compared to the control samples (*p* = 0.048), while no significant difference was observed between the late PE group and controls. Fragment size distribution was found to be significantly higher for the late-stage PE cases compared to controls (*p* = 0.036; Figure 1B), while fetal fraction was found to be significantly lower for the late-stage PE group compared to controls (*p* = 0.028; Figure 1C). In addition, linear regression plotting demonstrated that the use of fetal fraction and fragment size distribution could help distinguish between PE cases and control cases, apart from one outlier (case #85; Figure 1D). A linear regression plot for timepoint A with all three cfDNA signals is shown in Supplementary Figure 1.

**Figure 2:**
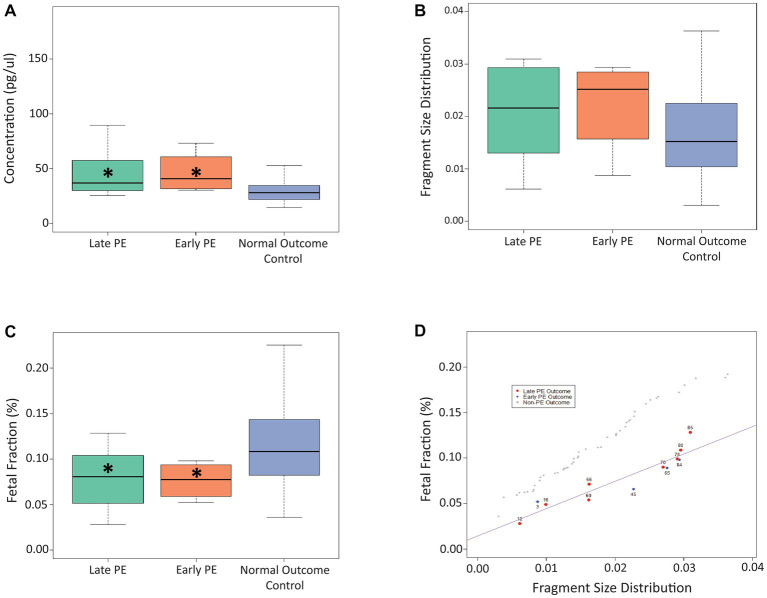
Use of cfDNA signals to distinguish between late PE, early PE, and control samples at timepoint B. **(A)** Use of concentration at timepoint B to distinguish between the three patient cohorts. **(B)** Use of fragment size distribution at timepoint B to distinguish between the three patient cohorts. **(C)** Use of fetal fraction at timepoint B to distinguish between the three patient cohorts. **(D)** Linear regression plot showing the use of fetal fraction and fragment size distribution to distinguish between PE samples and control samples at timepoint B. The Asterix (*) denotes significance between that particular PE group and the control group.

For timepoint B (17 + 6 to 25 + 5 weeks of gestation; Figure 2), we found that cfDNA concentration was significantly higher for both the early-stage (*p* = 0.021) and late-stage (*p* = 0.007) PE cases compared to controls, while the FF was significantly lower for both the early-stage (*p* = 0.044) and late-stage (*p* = 0.015) PE cases compared to controls. No significant differences were observed for fragment size distribution at timepoint B. Similar to timepoint A, linear regression plotting demonstrated that the use of fetal fraction and fragment size distribution could help distinguish between PE cases and control cases (Figure 2D). As can be seen, the outlier case (#85) from timepoint A aligned with the PE cases in timepoint B. A linear regression plot for timepoint B with all three cfDNA signals is shown in Supplementary Figure 2.

As a final step, we wanted to compare performance of our proposed model (based on the test set of data) at both timepoint A and timepoint B with the established CR model. As can be seen from Table 2, at timepoint A (first trimester of pregnancy), our model had a higher sensitivity (100% vs. 58.3%) and higher PPV (66.7% vs. 41.0%) compared to the CR model, with the same specificity observed for both models (87.5%).

**Table 2:** Summary of test performance for the cfDNA screening approach (*n* = 20) and the competing risk approach (*n* = 95).

Screening test for PE	Sensitivity, % (95% CI)	Specificity, % (95% CI)	False positives, n	False negatives, n	PPV, % (95% CI)
cfDNA approach (timepoint A)	100 (40–100)	87.5 (62.0–98.0)	2	0	66.7 (22–96)
cfDNA approach (timepoint B)	100 (40–100)	75.0 (48–93)	4	0	50.0 (16–84)
Competing risk approach	58.3 (28.0–85.0)	87.5 (79.0–94.0)	10	5	41.0

CI, confidence interval; PPV, positive predictive value.

## Discussion

In this proof-of-principle study we wanted to determine if cfDNA-based signals could be used to differentiate between patients that develop PE during pregnancy and those that do not. We found that analysis of cfDNA signals accurately identified patients who went on to develop PE, with a significant difference observed in concentration, fetal fraction, and fragment size distribution between PE patients and controls.

Previous studies have noted an association between increased fetal or total cfDNA concentration and preeclampsia. These increased levels of total and fetal cfDNA in patients with PE have been attributed to the accelerated apoptosis of trophoblastic cells due to placental ischemia and the impaired clearance of cfDNA from maternal blood (16, 18, 28). Lo et al. first demonstrated back in 1999 that the median concentration of circulating fetal DNA was much higher in preeclamptic patients than non-preeclamptic patients (18). The authors suggested that measurement of circulating DNA could possibly be a useful marker for diagnosis of PE. A 2005 study by Cotter et al. showed increased fetal DNA in the maternal circulation at 15 weeks in patients that went on to develop PE, and also noted a graded response between the quantity of fetal DNA and the severity of PE (29). A more recent study by Kolarova et al. found that total cfDNA was notably higher in preeclampsia at diagnosis, and that this higher total cfDNA correlated with an earlier gestational age at delivery and higher systolic blood pressure (19). Another study also found that PE was associated with higher maternal serum total cfDNA concentration than normal pregnancy (20). While a study by Rolnik et al. showed that there was a significant increase in median total cfDNA in early PE patients compared to controls, the authors found that measurements of total cfDNA at 11–13 weeks and 20–24 weeks of gestation were not predictive of PE (21). But none of these studies, to our knowledge, developed a new method for PE screening based on cfDNA and prospectively compared its head-to-head performance with the current reference method, the CR model, on the same population. In our study, cfDNA concentration was significantly higher for early-stage PE patients at timepoint A and significantly higher for both early-stage and late-stage PE patients at timepoint B, compared to control cases. We also found that the use of concentration in our logistic regression model was the least significant predictor of the three cfDNA parameters tested for timepoint A, but became quite significant and predictive for timepoint B. Our small sample size precluded delineating early PE patients from late PE for the model, but preliminary data suggests concentration may be of significant interest in future model development. Concentration dynamics between timepoint A and timepoint B warrants further exploration and understanding, as it may correlate with PE onset and would further inform about optimal timing for cfDNA screening.

Here, fetal fraction was significantly lower for the late-stage PE patients at timepoint A and significantly lower for both the early-stage and late-stage PE patients at timepoint B, compared to the patients that did not develop PE. Other studies have also shown that low FF is associated with PE (22–25). Rolnik et al. found a significant association between FF result and first-trimester markers for adverse pregnancy outcomes including PAPP-A and PlGF, with their results suggesting that patients at increased risk for PE tend to have lower fetal fractions (16). A study by Kolarova et al. hypothesized that the increase in total cfDNA in PE may be related to maternal tissue injury and the subsequent release of cfDNA from relevant organs (19). This could explain why there was not a similar corresponding rise in fetal cfDNA, which originates from the placenta, and thus why the FF is reduced.

Much research effort has targeted developing an easy and effective first-trimester PE screening tool. However, to date, no accurate single blood biomarker has been identified, particularly for prediction of late-onset PE (30). Although screening patients in the first trimester is optimal as it would allow implementation of timely prophylactic strategies for patients identified to be at risk of developing PE, we thought that it was also important to include a later timepoint (timepoint B) in our study. As some patients may not present for screening in their first trimester of pregnancy, we wanted to determine if our cfDNA-based model could be used to identify patients at risk of PE when screened later in their pregnancy. The established CR model for PE screening also allows a risk calculation based on maternal factors between 19 and 25 weeks of pregnancy (31). A number of other studies have also looked at biomarkers in the second trimester of pregnancy (32). A study by Kim et al. showed that the biomarker hypermethylated RASSF1A was more effective in the second and third trimesters of pregnancy compared to the first trimester (33). Previous studies have also shown that while PE screening in the first trimester can identify patients that will develop preterm PE, this approach is less effective at identifying patients that will develop PE at >37 weeks of gestation (32, 34). However, further screening in the second and third trimesters can help identify patients that develop term PE (35). Our approach also allowed us to examine the evolution of cfDNA prediction between two timepoints in pregnancy.

A strength of our study is the integration of multiple cfDNA signals into a novel PE screening tool. Our proposed cfDNA model appears to have improved sensitivity compared to the Competing Risk model, which is considered to be the gold standard of screening for PE, and could therefore be more effective. To our knowledge, this is the first study to report cfDNA fragment size data in the prediction of PE. Another strength of our proposed cfDNA model is that it is easy to use and so avoids a lot of the complications and special skills needed for other PE screening methods. It would also, in theory, be easy to incorporate into routine noninvasive prenatal screening. Future iterations of the model will likely expand the model to delineate early vs. late preeclampsia, in addition to including other key variables. As shown by our data from timepoint A, results could be available before 16 weeks of gestation which would allow for implementation of preventative treatment with aspirin as recommended by ACOG (2). However, a major limitation of our study is the small sample size, with only 12 PE patients in our study population. Future prospective studies would need to be carried out to determine the validation of our results in a larger patient population.

In conclusion, logistic regression modeling of preeclampsia outcomes with cfDNA fetal fraction, fragment size distribution, and concentration can assist with the probability prediction of pregnancies at risk for development of preeclampsia. The proposed model is theoretically a useful additional tool for screening, and subsequently counseling patients about risk and prophylaxis regarding the development of preeclampsia.

## Data availability statement

The datasets presented in this article are not readily available because of privacy or ethical restrictions. Requests to access the datasets should be directed to JG, jean.gekas.med@ssss.gouv.qc.ca.

## Ethics statement

The studies involving human participants were reviewed and approved by the Prenato Clinics Institutional Research Review Board. The patients/participants provided their written informed consent to participate in this study.

## Author contributions

JG and SB contributed to the conceptualization, methodology of the study, and developing the study protocol. JG and M-AR contributed to the study protocol, consent, and ethics approval. TB supported the cfDNA analysis and modeling of the data. KJ contributed to the interpretation of the data and supported literature review and drafting of the manuscript. All authors were involved in manuscript development, editing, and review of the manuscript, and have read and approved the manuscript.

## Funding

This study was funded by Illumina, Inc.

## Conflict of interest

TB and SB are employees of and own equity in Illumina, Inc. KJ is a paid consultant for Illumina, Inc. JG and M-AR hold positions at Quebec University Hospital but collaborate respectively as medical and scientific advisors at Prenato clinics, a private medical clinic network in Quebec province, Canada. This study received funding from Illumina, Inc. The funder had the following involvement with the study: study design, analysis and interpretation of data, writing of the article, and the decision to submit the article for publication. All authors declare no other competing interests.

## Publisher’s note

All claims expressed in this article are solely those of the authors and do not necessarily represent those of their affiliated organizations, or those of the publisher, the editors and the reviewers. Any product that may be evaluated in this article, or claim that may be made by its manufacturer, is not guaranteed or endorsed by the publisher.
