# Supplementary material for: Use of cell-free signals as biomarkers for early and easy prediction of preeclampsia
Source: Front Med (Lausanne). 2023 May 24;10:1191163. doi: 10.3389/fmed.2023.1191163 (PMC10244626; doi:10.3389/fmed.2023.1191163)

Supplementary Figure 1. Linear regression analysis for timepoint A using cfDNA concentration, fetal fraction, and fragment size distribution.

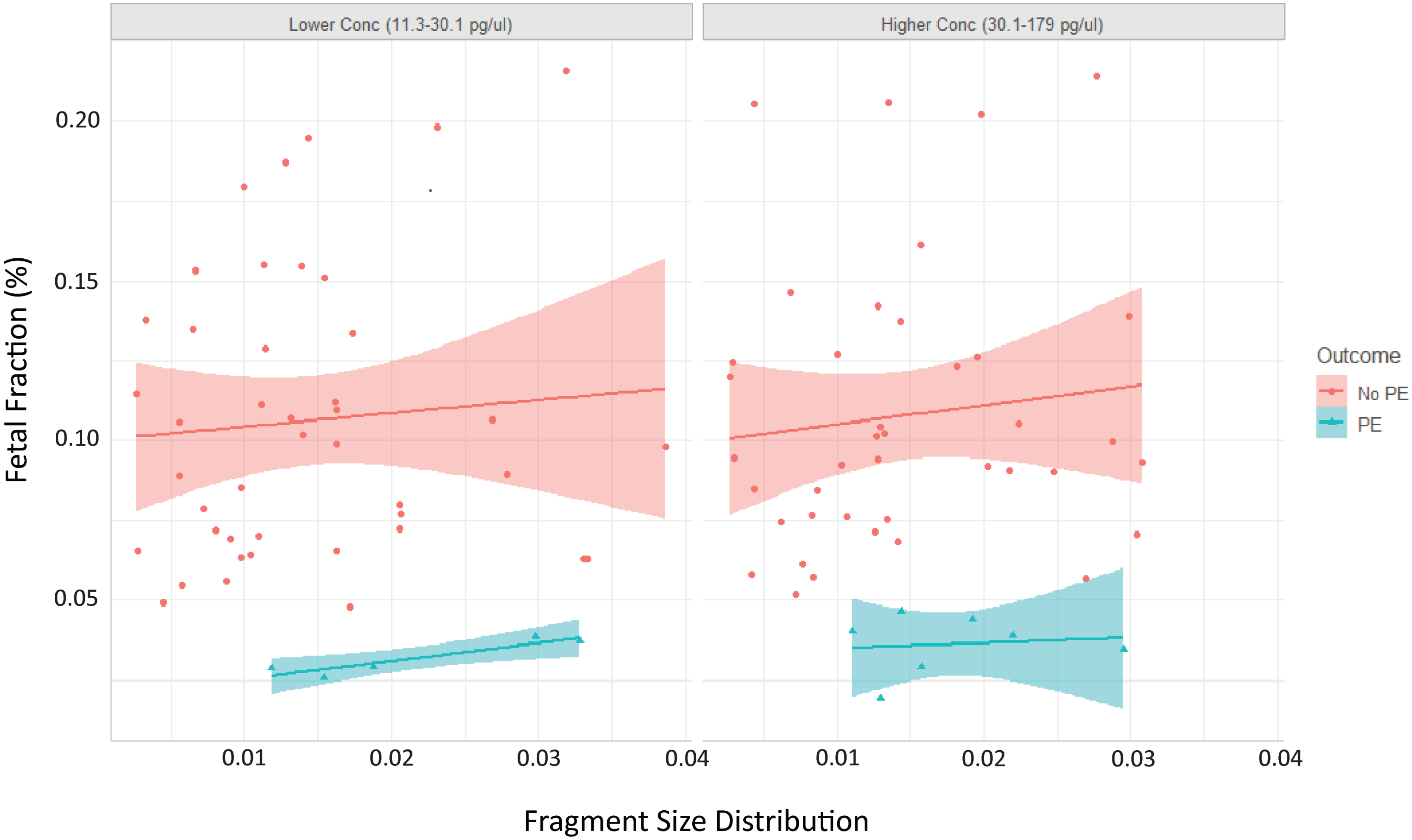

Supplement: Supplementary file 1 [file Image_1.PDF]
